# Landmarks to guide femoral insertion in lateral patellofemoral ligament reconstruction: An in vivo assessment of isometry

**DOI:** 10.1002/ksa.12635

**Published:** 2025-02-24

**Authors:** Miriam R. Boot, Sebastiaan A. W. van de Groes, Esther Tanck, Dennis Janssen

**Affiliations:** ^1^ Orthopaedic Research Laboratory, Department of Orthopaedics Radboud University Medical Center Nijmegen The Netherlands

**Keywords:** dynamic computed tomography, femoral tunnel positioning, in vivo, isometry, lateral patellofemoral ligament (LPFL)

## Abstract

**Purpose:**

Lateral patellofemoral ligament (LPFL) reconstruction addresses medial patellar instability, but uncertainty regarding the optimal femoral attachment site may affect isometry and increase complication rates. This study aimed to establish landmarks for the femoral attachment of the LPFL graft based on in vivo isometry during active knee extension.

**Methods:**

Dynamic computed tomography scans of 104 knees from 58 healthy participants were employed to examine flexion‐extension movements. Length changes were assessed in approximately 1335 virtual LPFL graft fibres, which extended from the proximal one‐third of the patellar height to attachments across the femoral condyle. Four methods were evaluated for achieving (near‐)isometric LPFL graft behaviour: three radiographic methods (R1–R3) and one anatomic method (A). Method R1 positioned the femoral attachment at a lateral equivalent of Schöttle's point, Method R2 at the centre of the trochlear groove arc, Method R3 at the centre of the lateral trochlear ridge arc and Method A at a point relative to the lateral epicondyle.

**Results:**

Median length changes during extension were 7.7 mm (Method R1), 3.4 mm (Method R2), 2.7 mm (Method R3) and 3.0 mm (Method A). Method R3 demonstrated significantly smaller length changes compared to Methods R1 (*p* < 0.001) and R2 (*p* < 0.01), while Method A yielded smaller changes than Method R1 (*p* < 0.001). Notably, Method R1 resulted in continuous LPFL graft tightening throughout knee motion, whereas Methods R2, R3 and A showed initial tightening until 20° flexion, followed by slackening and near‐isometric behaviour.

**Conclusion:**

Femoral graft attachment is best determined at the centre of the lateral trochlear ridge arc (Method R3) or 15.1 mm anterior and 3.4 mm proximal to the lateral epicondyle (Method A). These guidelines help improve surgical precision and minimize complications in LPFL reconstructions.

**Level of Evidence:**

Level III.

**Abbreviations**
3D: three‐dimensional
A: 15.1 mm anterior and 3.4 mm proximal to the lateral epicondyle
AP: anterior‐posterior
CDI: Caton–Deschamps index
CT: computed tomography
IQR: interquartile range
LPFL: lateral patellofemoral ligament
LTI: lateral trochlear inclination
ML: medial‐lateral
MPFL: medial patellofemoral ligament
PD: proximal‐distal
R1: lateral equivalent of Schöttle's point
R2: the centre of an arc formed by the trochlear groove line
R3: the centre of an arc formed by the lateral trochlear ridge
ROI: region of interest
SD: standard deviation
TT‐TG: tibial tuberosity–trochlear groove

## INTRODUCTION

Medial patellar instability is a rare but debilitating condition, almost exclusively caused as an iatrogenic complication following inappropriate lateral release procedures, though it can also result from other causes, including trauma [30]. Patients with this condition frequently experience knee pain, medial (sub)luxations, medial apprehension, and swelling, impacting daily activities [2, 23, 30]. Lateral patellofemoral ligament (LPFL) reconstruction has shown promising clinical outcomes in restoring knee stability in several case series [2, 5, 22, 27, 32]. However, surgical variability [11] and the absence of standardized guidelines continue to pose major challenges.

Accurate femoral attachment placement is a critical aspect of LPFL reconstruction. Studies on medial patellofemoral ligament (MPFL) reconstructions have shown that incorrect graft placement can alter knee kinematics, disrupt pressure distribution and overload the graft, which can lead to pain, recurrent instability and patellofemoral osteoarthritis [3, 24, 26]. To enhance femoral graft positioning in MPFL reconstruction, Schöttle et al. [28] identified radiographic landmarks that improved surgical accuracy [16]. Given the similar biomechanical role of LPFL reconstruction in preventing recurrent patellar dislocations and restoring knee stability, identifying reliable landmarks for femoral attachment could similarly enhance surgical accuracy and improve patient outcomes.

To date, research on LPFL reconstruction remains limited, largely due to the rarity of the procedure. Existing studies identified the centre of the anatomical LPFL attachment relative to landmarks, but these have been based on small sample sizes (*n* = 10 cadaveric specimens per study) and have not specifically addressed isometric graft positioning [13, 21, 29]. Since isometric graft placement is typically pursued in LPFL reconstructions and is considered important for optimizing knee stability [2, 5, 27, 32], larger studies are needed to establish landmarks facilitating optimal femoral attachment positioning from an isometric perspective.

This study aimed to establish reliable radiographic and anatomical landmarks for selecting the optimal femoral attachment site in LPFL reconstruction, ensuring consistent near‐isometric graft behaviour. Defining these landmarks enhances intraoperative guidance and improves postoperative evaluation, ultimately leading to greater surgical precision and improved patient outcomes. The hypothesis was that a distinct femoral attachment site existed, achieving near‐isometric graft behaviour and being consistently identifiable using radiographic and anatomical landmarks.

## MATERIALS AND METHODS

### Participants

**Figure 1:**
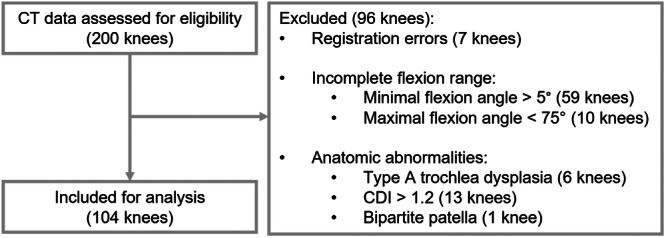
Participant inclusion flowchart. CDI, Caton–Deschamps index; CT, computed tomography.

A data set was used comprising static and dynamic computed tomography (CT) scans of 200 knees from 100 healthy participants aged 18–35 years with no previous knee surgeries, knee injuries, knee complaints, functional or congenital disorders, or visible varus or valgus malalignment [9]. Institutional review board approval was obtained, and all participants provided written informed consent for secondary use of their data. Of these 200 knees in the data set, 104 knees from 58 participants were included (Table 1). The inclusion and exclusion criteria for this study largely follow those established in our previous publication and are summarized in Figure 1 [4]. Knees were excluded if the CT scans did not adequately capture knee anatomy due to image artefacts or incorrect positioning within the scanner, resulting in the loss of positional information for either of the bones. Other exclusion criteria included an incomplete range of knee flexion, defined as a minimal flexion angle >5° or a maximal flexion angle <75°, to ensure that only knees with a sufficient range of motion were included in the study. Additionally, knees with anatomical abnormalities were excluded, including Type A trochlear dysplasia, a Caton–Deschamps Index (CDI) >1.2, a tibial tuberosity‐trochlear groove (TT‐TG) distance >20 mm, or a bipartite patella.

**Table 1:** Participant and knee characteristics.

	Value
Participant characteristics (*N* = 58)	
Age, years	24.3 ± 3.3 (19–34)
Male:female sex, *n*	11:47
Length, cm	171.8 ± 7.5 (158–190)
Weight, kg	66.0 ± 8.7 (54–98)
Body mass index	22.4 ± 2.7 (17.7–34.3)
Knee characteristics (*N* = 104)	
Right:left side, *n*	54:50 (46 pairs)
CDI	1.0 (0.9–1.1) [0.7–1.2]
TT‐TG distance, mm	12.5 (10.6–14.2) [0.2–19.1]
LTI, deg	19.7 (17.7–22.8) [11.4–28.6]

*Note*: Data are shown as mean ± SD (range) or median (interquartile range) [range] unless otherwise indicated.

Abbreviations: CDI, Caton–Deschamps index; LTI, lateral trochlear inclination; SD, standard deviation; TT‐TG, tibial tuberosity–trochlear groove.

### Procedures

Image acquisition and construction of bone models have already been described in detail [9]. Static CT scans (Aquilion ONE CT scanner, Canon Medical Systems Europe B.V.) were conducted while participants lay in supine position (field of view, 50 cm; mean voxel size: 0.71 × 0.71 × 0.80 mm^3^). Dynamic CT scans were conducted during a flexion‐extension‐flexion movement between 90° and full extension over 10 s, acquiring 41 CT frames (field of view, 16 cm; mean voxel size: 0.98 × 0.98 × 0.50 mm^3^). Participants practised the movement prior to image acquisition. Nonetheless, some participants started knee movement prematurely, failed to achieve full leg extension, or executed the movement too slowly, resulting in an incomplete flexion‐extension‐flexion range. This was addressed by only including participants who achieved an extension range from ≥75° to ≤5°. The CT data were used to construct bone models for each femur, patella and tibia. First, both knees were segmented using a U‐Net convolutional neural network architecture (dice similarity coefficients; femur: 0.99; patella: 0.96; tibia: 0.98) [20]. Then, the bone masks were converted to three‐dimensional (3D) surface meshes using MATLAB (Version R2021b; MathWorks). The static bone models were registered to each dynamic bone model of the same knee using point set registration with a coherent point drift algorithm succeeded by intensity‐based B‐spline rigid registration using the elastix toolbox [19]. The accuracy of these steps was approximately 1.0° and 1.0 mm for rotation and translation, respectively [9]. Furthermore, anatomical coordinate systems were assigned to obtain the medial‐lateral (ML), anterior‐posterior (AP) and proximal‐distal (PD) axis of the femur, tibia and patella [7]. Afterwards, bone models from dynamic CT frames between 75° and 80° of flexion and full knee extension (closest to 0°) were selected for LPFL graft fibre measurement, resulting in approximately 18 measurements. Focus was placed on the extension phase to reduce computational time with little compromise on accuracy, given the similarity in the knee kinematics [9] and MPFL length changes [4] during extension and flexion.

### Patellofemoral attachments

**Figure 2:**
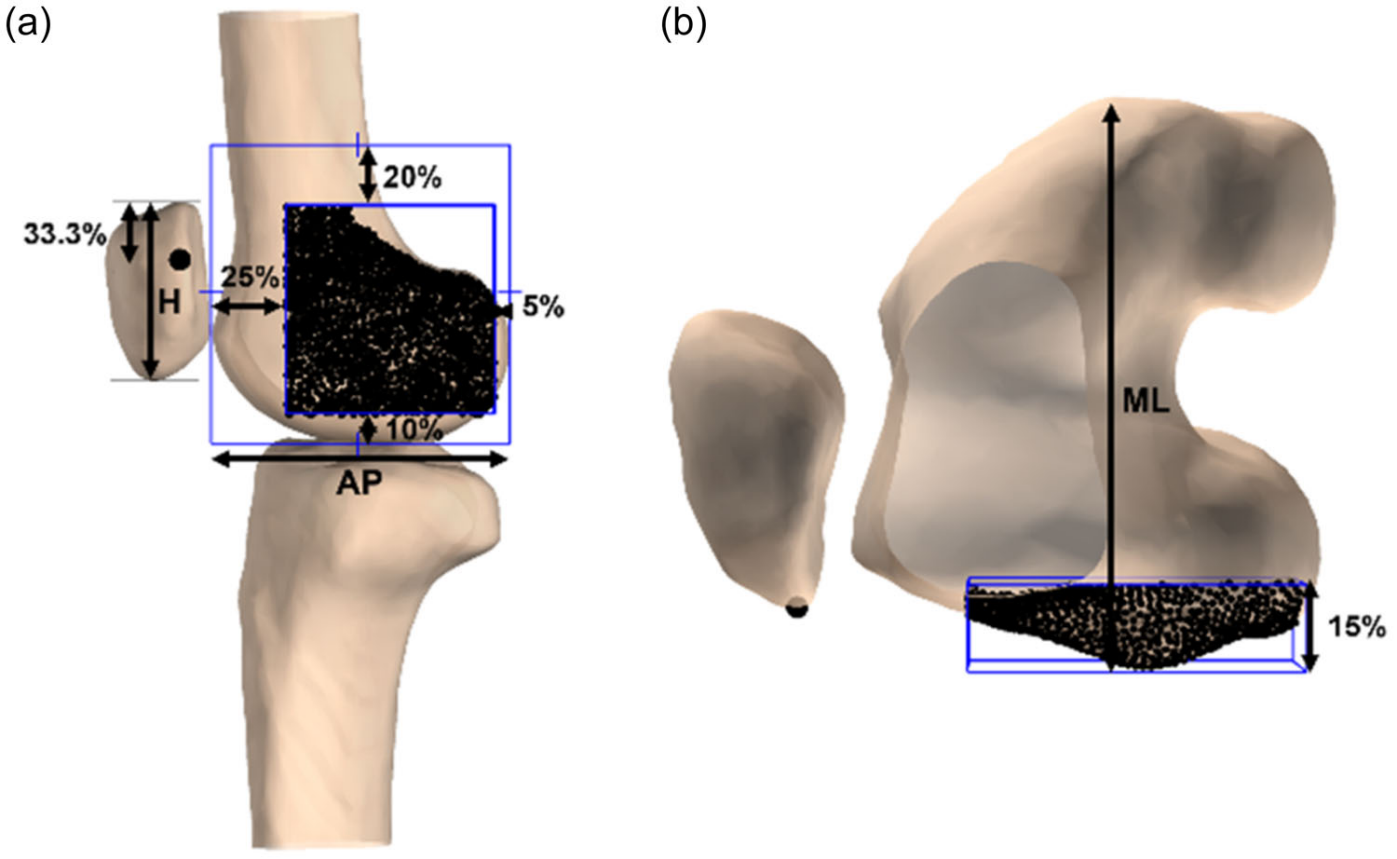
Lateral view of a left knee in extension showing the patellofemoral attachments of lateral patellofemoral ligament (LPFL) graft fibres. (a) The most lateral point at the proximal one third of the sagittal patellar height (H) was selected as patellar attachment (black dot) and a median of 1335 points within a region of interest (ROI) as femoral attachments. The anterior‐posterior (AP) and proximal‐distal (PD) distances of the ROI were scaled to the AP width of the lateral condyle. (b) The depth of the ROI was scaled to the medial‐lateral (ML) width of the distal femur.

At the femoral side, attachment points were selected within a region of interest (ROI) encompassing the LPFL graft site [2, 5, 27, 32] and the anatomical LPFL origin [6, 13, 21, 29]. To select these points, the femur was remeshed to an average element size of 1.0 mm and rotated to a lateral view with the PD axis aligned to the posterior cortex extension line. The ROI was scaled based on the AP distance of the lateral condyle and the maximal femoral ML width to accommodate variations in knee size (Figure 2). At the patellar side, the most lateral point was selected at the proximal one third of the sagittal patellar height in extension (Figure 2). This selection was based on anatomical studies, ensuring it fell within the known patellar attachment width of the LPFL [13, 21, 29]. Ultimately, 1335 LPFL graft fibres (interquartile range [IQR], 1219–1428) were created.

### Length change measurement

**Figure 3:**
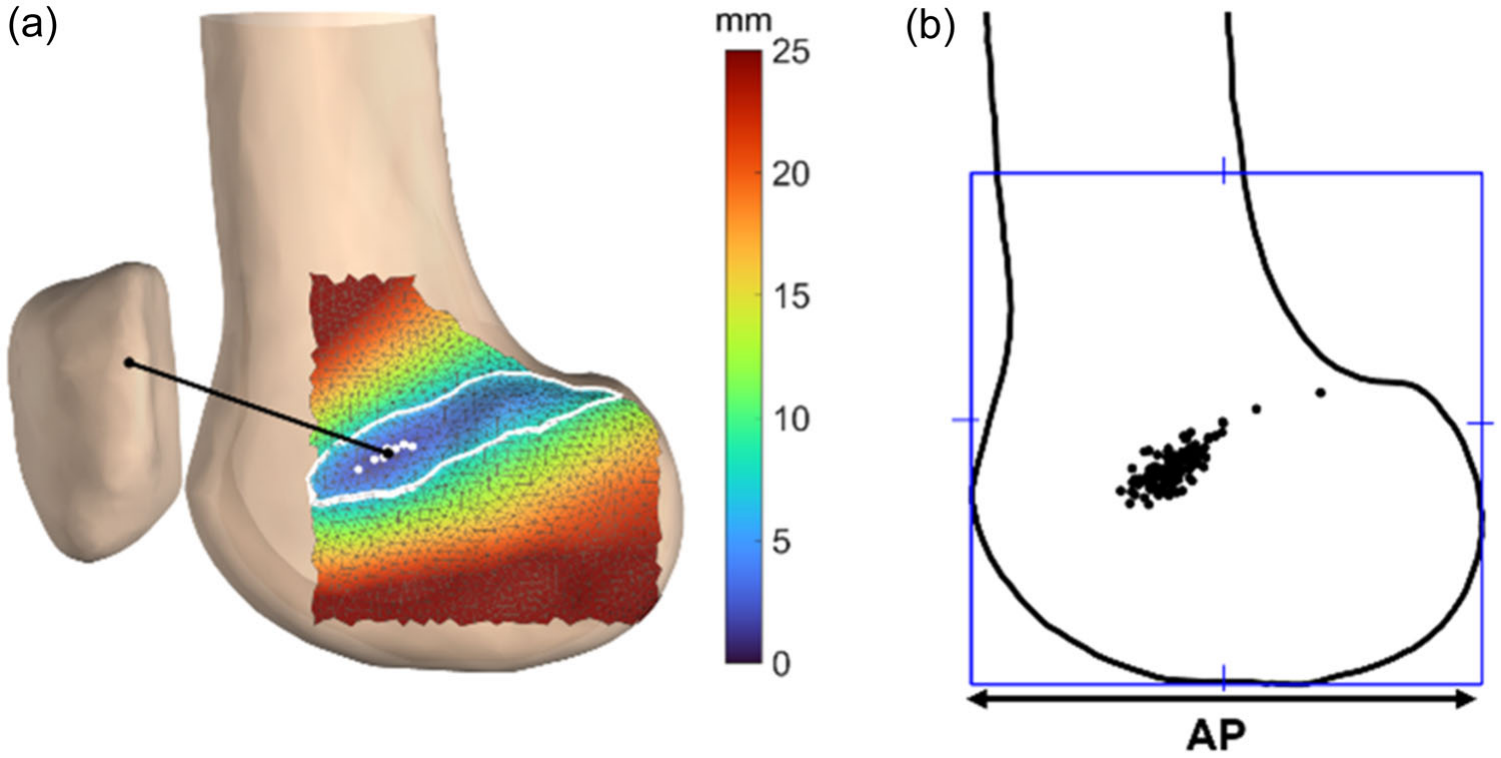
(a) Heat map illustrating a typical distribution of lateral patellofemoral ligament graft length changes (maximal length − minimal length) in millimetres (mm) of a left knee during extension. The 20.0% of virtual graft fibres with the smallest overall length change are encircled in white. The (near‐)isometric area is indicated with white dots. The graft fibre from the centre of the (near‐)isometric area to the patellar attachment is indicated in black and referred to as the most isometric point. (b) The most isometric point for each knee is displayed on the left knee. Each point is scaled relative to a square with sides equal to the anterior‐posterior distance of the lateral condyle.

Virtual LPFL graft fibres were generated by projecting the line between the patellar and femoral attachments onto the lateral femoral condyle surface, using convex hull calculations to account for ligament wrapping. Afterwards, an optimization procedure was applied to the 20.0% of virtual graft fibres with the smallest length changes to determine the shortest convex hull path (Figure 3) [1]. The (near‐)isometric graft fibres were then identified and defined as those with a length change of ≤2.0 mm, consistent with the estimated measurement accuracy. This resulted in a small area with a median of six graft fibres (IQR, 2–12). The centre of this area in the AP and PD directions was defined as the most isometric point. LPFL graft fibre length changes were assessed in all 104 knees, identifying the most isometric point for each knee (Figure 3).

### Selection of landmarks

**Figure 4:**
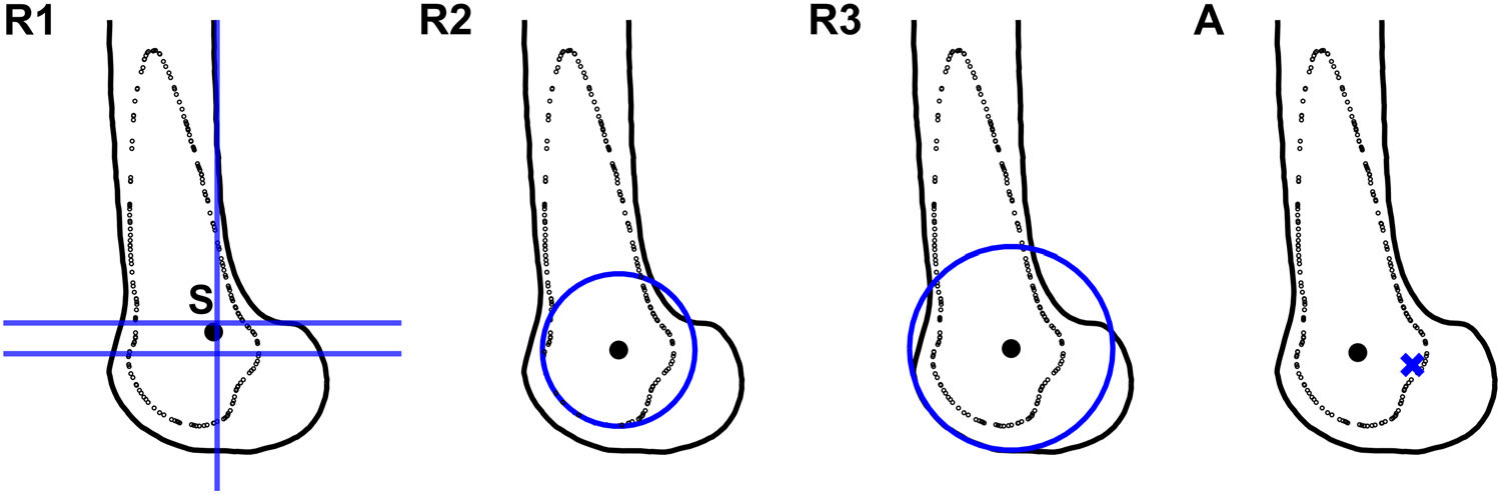
Methods for delineating the femoral graft attachment site of the lateral patellofemoral ligament (LPFL) graft on a left knee. Four methods are illustrated: three methods using radiographic landmarks (R1, R2 and R3) and one method using an anatomical landmark (A). Method R1 utilizes the lateral equivalent of Schöttle's point (S), located 1.0 mm anterior to the posterior cortex extension line (vertical line) and 2.5 mm distal to a perpendicular line that intersects the posterior origin of the lateral femoral condyle (upper horizontal line). For clarity, the perpendicular line intersecting the most posterior point of the Blumensaat line is also shown (lower horizontal line). Method R2 describes the femoral attachment as the centre of an arc formed by the trochlear groove line. Method R3 describes the femoral attachment as the centre of an arc formed by the lateral trochlear ridge. Method A describes the femoral attachment 15.1 mm anterior and 3.4 mm proximal to the lateral epicondyle (blue cross).

After identifying the most isometric attachment for each knee, the effectiveness of three radiographic methods (R1–R3) and one anatomic method (A) was evaluated based on LPFL graft length changes (Figure 4). Method R1 utilized the lateral equivalent of Schöttle's point, positioned 1.0 mm anterior to the posterior cortex extension line and 2.5 mm distal to a perpendicular intersecting the posterior origin of the lateral femoral condyle [28]. Method R2 defined femoral graft attachment using a circle fitted to the trochlear groove line using a least‐squares approach, reflecting its circular sagittal geometry [10, 15]. Similarly, Method R3 utilized a circle fitted to the lateral trochlear ridge. The circle centres served as the graft attachment points for both methods, as the patella follows the geometry of the distal femur [14], suggesting the circle centre as a reasonable estimate for the most isometric point. Method A placed a point 15.1 mm anterior and 3.4 mm proximal to the lateral epicondyle, corresponding to the median distance from the most isometric attachment, with the lateral epicondyle defined as the most lateral point of the distal femur. Afterwards, LPFL graft length changes were measured at each reference point, selecting the method with the smallest overall length change as the preferred approach.

### Statistical analysis

The primary outcome was to identify optimal radiographic or anatomic landmarks for selecting an LPFL graft with minimal length changes. The graft length changes at the reference points obtained with Methods R1–R3 and A did not meet the assumption of normality, as evidenced by histograms and Shapiro–Wilk tests, and were therefore presented as median values with the IQR. Friedman tests with Tukey post hoc analyses were employed to detect significant differences in overall length change between the methods. In cases of statistical significance, preference was given to the method associated with the smallest graft length changes. Seventeen outliers were identified using the IQR method (data points below Q1 − 1.5IQR or above Q3 + 1.5IQR). These outliers were visually reviewed by an experienced orthopaedic surgeon (S.vdG.), confirming the absence of major anatomical or kinematic abnormalities justifying exclusion. Furthermore, sensitivity analysis demonstrated that the exclusion of these outliers had no impact on significance tests. Statistical analyses were performed using MATLAB, with significance set at *p* < 0.05.

## RESULTS

**Figure 5:**
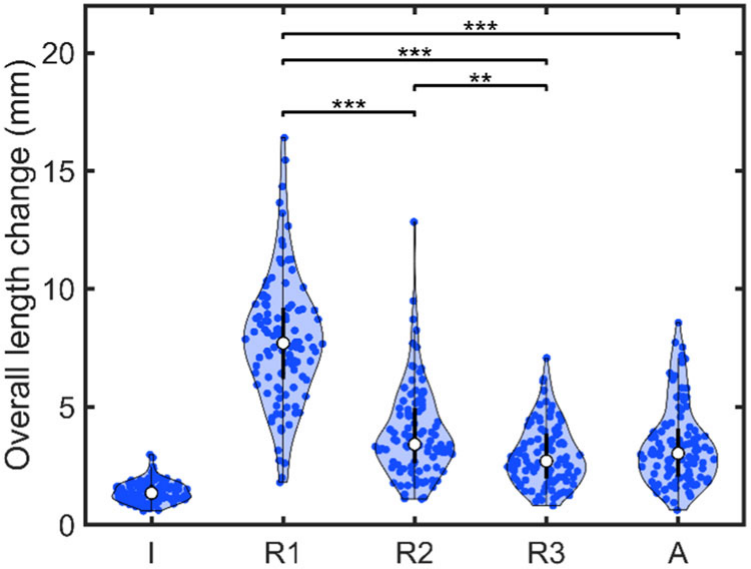
Violin plots showing the overall lateral patellofemoral ligament graft length change during knee extension at the most isometric attachment (I), the radiographic points obtained with Methods R1 through R3, and the anatomic point obtained with Method A (see Figure 4). Blue dots indicate individual data points. Asterisks indicate significant differences between the methods, **p < 0.01, ***p < 0.001.

Figure 5 presents the overall LPFL graft length change during knee motion. As Figure 5 shows, the median overall length change was 1.4 mm (IQR, 1.1–1.7 mm) for the most isometric attachment, 7.7 mm (IQR, 6.2–9.2 mm) for Method R1 (i.e., at the lateral equivalent of Schöttle's point), 3.4 mm (IQR, 2.7–5.0 mm) for Method R2, 2.7 mm (IQR, 2.0–3.9 mm) for Method R3 and 3.0 mm (IQR, 2.1–4.1 mm) for Method A. The median graft length at the most isometric attachment was 34.9 mm (IQR, 33.1–37.4 mm) in full extension.

**Figure 6:**
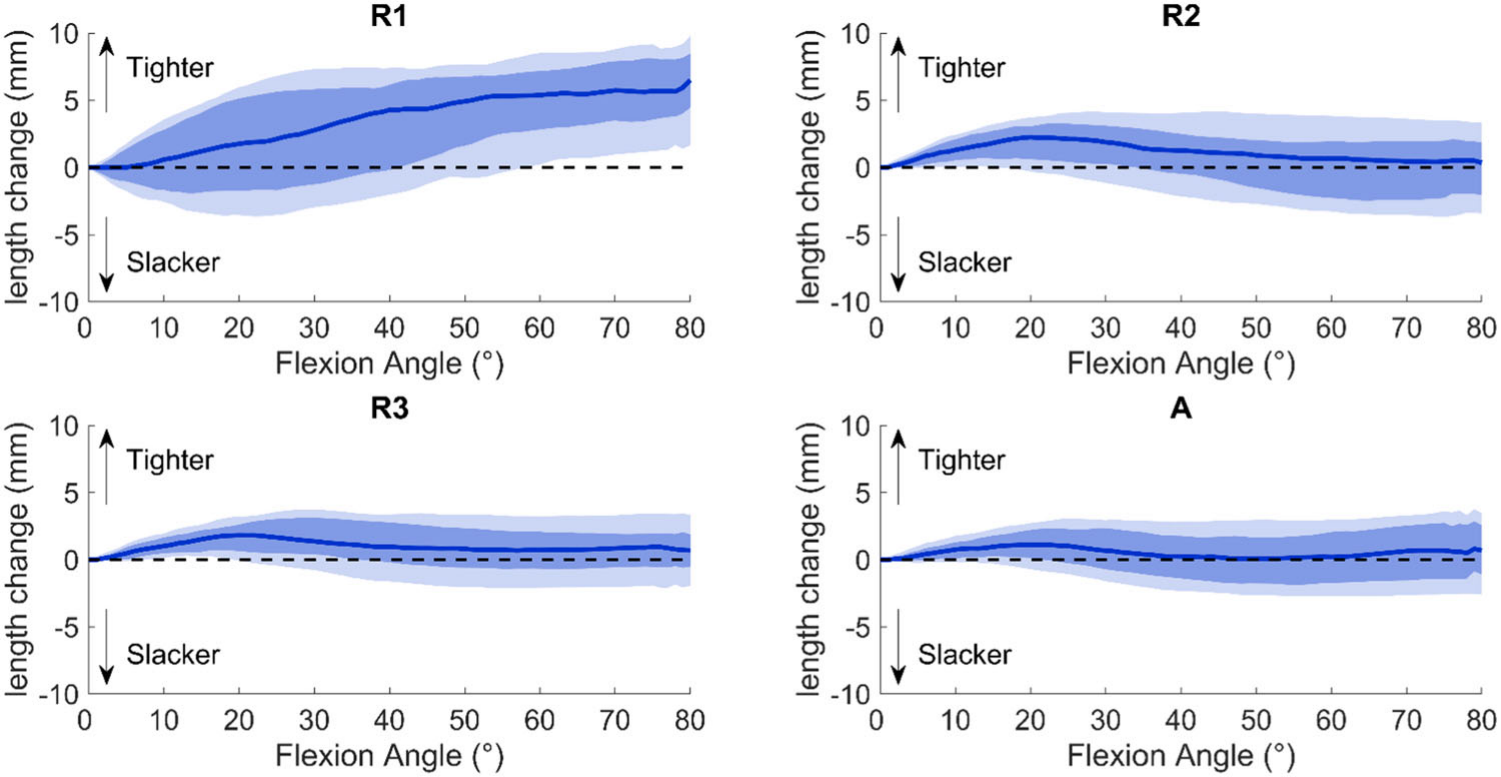
Lateral patellofemoral ligament (LPFL) graft length changes (mm) relative to full extension between 0° and 80° for three radiographic methods (R1 through R3) and one anatomic method (A) (see Figure 4). A decrease indicated LPFL graft slackening, whereas an increase indicated LPFL graft tightening. The data were obtained during knee extension but are displayed from 0° to 80° to facilitate comparison with existing literature. Solid lines represent interparticipant medians, dark shading represents the interquartile range (IQR), and light shading represents ±1.5 IQR (12.5th–87.5th percentiles). The horizontal dotted line represents isometric behaviour of the LPFL graft. The data between 0°–5° and 75°–80° of flexion represent a subset of participants (38% reached 0° flexion, 53% reached 80° flexion).

Statistical testing showed that Method R1 had a significantly greater length change compared to Methods R2, R3 and A (*p* < 0.001), and Method R2 had a greater length change compared to Method R3 (*p* < 0.01). The variability in overall length change was also higher for Method R1 compared to Methods R3 and A. Accordingly, the trends in LPFL graft length changes per flexion angle differed notably between Method R1 and Methods R2, R3 and A (Figure 6). With Method R1, the median graft length continuously increased with flexion, indicating continuous tightening. Conversely, Methods R2, R3 and A showed an initial tightening of the median length up to 20° of flexion, followed by a gradual restoration toward extension length, stabilizing around 40° of flexion.

## DISCUSSION

The most important findings of this study were that the LPFL graft length changes were significantly smaller with Method R3 compared to the other radiographic methods (R1 and R2) and were comparable to the anatomic method (A). The median overall length change between 0° and 80° of flexion was 2.7 mm for Method R3, 7.7 mm for method R1, 3.4 mm for Method R2 and 3.0 mm for Method A. These novel findings indicate that the femoral attachment can be determined radiographically as the centre of an arc formed by the lateral trochlear ridge (Method R3), or anatomically at a point 15.1 mm anterior and 3.4 mm proximal to the lateral epicondyle (Method A).

**Figure 7:**
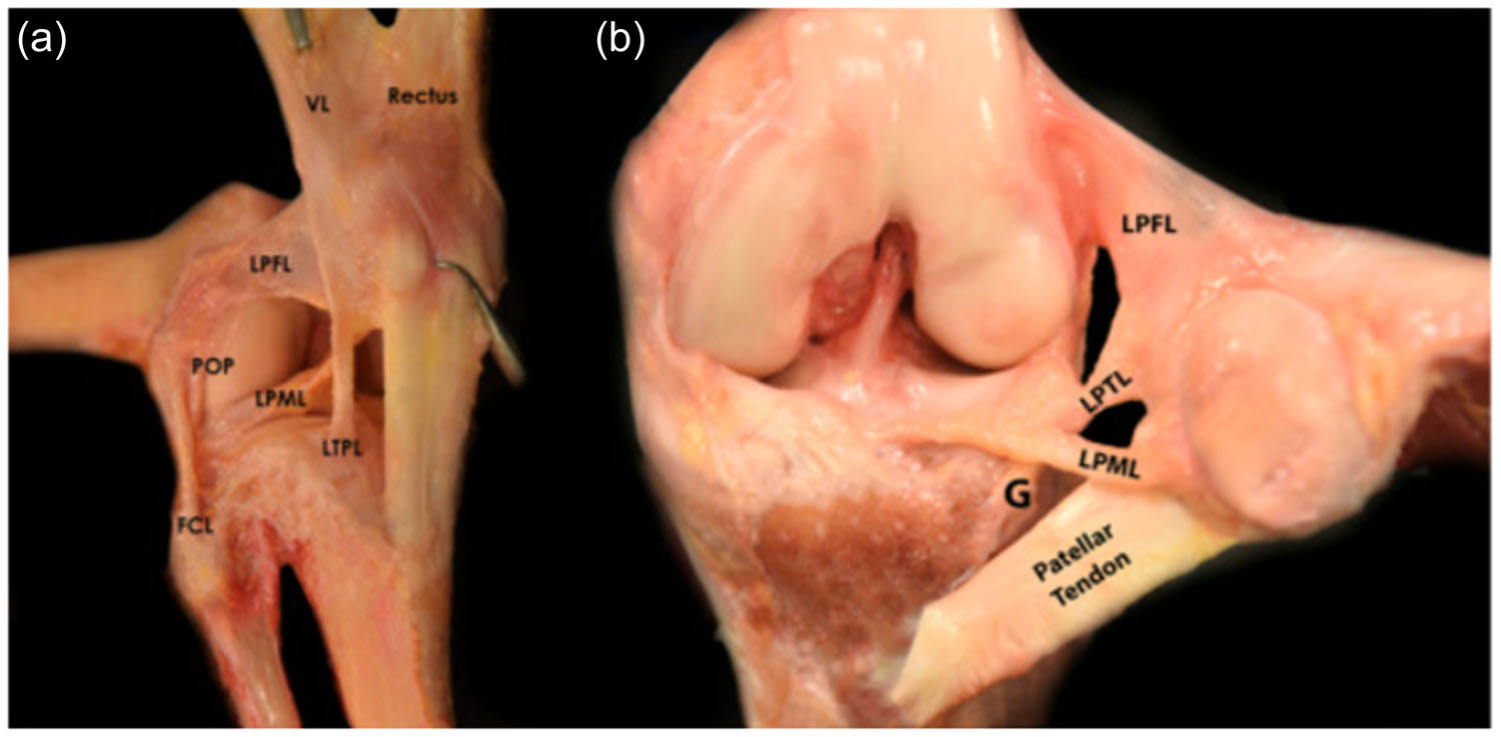
Lateral patellofemoral complex and associated structures. (a) Cadaveric dissection of a right knee demonstrating the critical soft tissue structures to the lateral patellofemoral complex (LPFC), including the lateral patellofemoral ligament (LPFL), lateral patellomeniscal ligament (LPML) and lateral patellotibial ligament (LPTL). Also shown is the anatomic relationship of the vastus lateralis (VL), rectus femoris (Rectus), fibular collateral ligament (FCL), and popliteus tendon (POP) to the LPFC. (b) Cadaveric dissection of the left knee of the LPFL, LPML and LPTL in relation to Gerdy's (G) tubercle. Reprinted from Operative Techniques in Sports Medicine, Vol. 31, Issue 4, Kerzner B, Kaplan DJ, Fortier LM, Khan ZA, McCormick JR, Hinckel BB, Chahla J, Lateral Patellofemoral Ligament Reconstruction: Anatomy, Biomechanics, Indications, and Surgical Techniques, page 151036, Copyright (2023), with permission from Elsevier.

To date, no studies have defined the optimal femoral attachment for obtaining (near‐)isometric LPFL graft placement. Instead, prior studies have focused on the anatomical LPFL attachment centre in cadaveric specimens [13, 21, 29]. Huddleston et al. [13] reported an average LPFL length change of 11.6 mm during knee flexion, considerably exceeding the median length change of 1.4 mm at the most isometric attachment in this study. These findings suggest that the anatomical centre may not be ideal for achieving graft isometry. Given that the LPFL is part of a complex that also includes the lateral patellomeniscal ligament and the lateral patellotibial ligament (Figure 7), interactions between these ligaments may cause non‐isometric behaviour of the anatomical centre of the LPFL attachment. Alternatively, the observed differences in length change might result from variations in measurement methodology. Huddleston et al. [13] used quasi‐static passive knee flexion with a 1‐kg load to simulate quadriceps tension, which may not reflect dynamic in vivo flexion‐extension movements. Additionally, they measured direct distances between attachment points without accounting for ligament wrapping, potentially contributing to length change differences.

In contrast to the limited research on LPFL length changes, MPFL length studies show that excessive graft tightening during knee flexion can lead to excessive constraint on the patellofemoral joint during deep flexion, causing pain and failure [3, 26]. In this study, the LPFL graft tightened continuously throughout knee flexion at the lateral equivalent of Schöttle's point (Method R1). In contrast, it slackened after 20° and stabilized around 40° of flexion for the other methods (R2, R3 and A). This reinforces that the lateral equivalent of Schöttle's point is unsuitable for LPFL graft placement. Furthermore, our findings support tensioning the graft at 20° of knee flexion as the optimal fixation angle, aligning with the principle that grafts should be fixated at the flexion angle with maximal graft tension [12].

This study provides novel insights into radiographic and anatomic landmarks for guiding femoral attachment selection. Anatomical studies reported mean distances of 10.8–11.5 mm anteriorly and 2.6–7.1 mm distally from the anatomical centre to the lateral epicondyle [13, 29], whereas this study found a median distance of 15.1 mm anteriorly and 3.4 mm proximally from the most isometric attachment to the lateral epicondyle. This finding suggests that the most isometric attachment is located more anteriorly and proximally compared to the anatomical centre. However, these differences may also stem from individual anatomical variations. Further research is needed to clarify these differences and their impact on postoperative outcomes to understand their clinical importance.

Although LPFL reconstructions are rare, our findings contribute to reducing complications from incorrect femoral graft attachments in LPFL reconstructions by providing clinically applicable landmarks to enhance both intraoperative guidance and postoperative evaluation. Specifically, method R3 offers an easily identifiable intraoperative radiographic landmark, which can be located using, for example, fluoroscopy, similar to Schöttle's point for MPFL reconstructions. Method A presents a practical alternative that can be located through palpation of the lateral epicondyle. Postoperatively, the radiographic landmark allows for straightforward verification of graft placement by drawing a circle through the lateral trochlear ridge on lateral radiographs. Further research in clinical populations is warranted to validate the effectiveness of these methods in patients with medial patellar instability.

Several limitations should be noted. First, our study only measured LPFL graft isometry at the proximal one third of the sagittal patellar height, so our findings may not fully represent LPFL reconstructions involving different patellar graft fixation points. However, it has been shown for the MPFL that varying the patellar attachment has only a minor effect on MPFL length changes compared to varying the femoral attachment [18, 31]. This might imply that varying the patellar attachment will also have a minor impact on the most isometric attachment of the LPFL graft. Second, data were collected during a non‐weightbearing, open‐kinetic chain active flexion‐extension movement, so our results may not represent different daily activities [17, 25]. Nevertheless, this is the first study to examine LPFL graft length changes in vivo during active knee motion, making our measurements more realistic than those reported in the literature [13]. Third, only healthy knees were investigated, so the applicability of the radiographic and anatomic methods cannot be guaranteed in patients with medial instability, who typically suffer from morphological abnormalities, including trochlear dysplasia [3, 8]. These abnormalities particularly may affect the applicability of Methods R2 and R3, as these methods define the reference points based on the trochlear groove line and lateral trochlear ridge, respectively. However, by studying healthy knees, LPFL graft isometry and the most isometric reference points could be assessed under normal knee kinematics, providing direct guidelines for both normal knees and those with corrected abnormalities.

Despite these limitations, our study has yielded valuable methods for intraoperative selection and postoperative evaluation of the most isometric femoral graft attachment. Although LPFL reconstruction is a rare procedure, typically necessitated by improper lateral release, these findings offer important guidance for improving surgical outcomes. With a sample size of 104 knees, this study surpasses previous research in defining a reference point to guide LPFL reconstructions. Clinically, these findings enable more accurate graft placement by providing easy‐to‐use guidelines for intraoperative assessment using radiographic or anatomic landmarks. These methods help to improve patient outcomes by reducing the risk of graft complications, such as overtightening, and facilitate postoperative evaluation.

## CONCLUSION

Femoral attachment in LPFL reconstructions can be guided radiographically by using the centre of an arc formed by the lateral trochlear ridge (Method R3), or anatomically by selecting a point 15.1 mm anterior and 3.4 mm proximal to the lateral epicondyle (Method A). In contrast, the lateral equivalent of Schöttle's point (Method R1) is unsuitable for guiding femoral attachment in LPFL reconstructions due to excessive length changes and continuous tightening during knee flexion. Further research is warranted to confirm the effectiveness of these radiographic and anatomic approaches in clinical settings.

## AUTHOR CONTRIBUTIONS

*Conceptualization, methodology and data analysis*: Sebastiaan A. W. van de Groes, Dennis Janssen and Miriam R. Boot. *Writing—original draft preparation*: Miriam R. Boot. *Writing—review and editing*: Sebastiaan A. W. van de Groes, Dennis Janssen and Esther Tanck.

## CONFLICT OF INTEREST STATEMENT

The authors declare no conflicts of interest.

## ETHICS STATEMENT

This study was performed in line with the principles of the Declaration of Helsinki. Approval was granted by the Medical Ethical Committee of the Radboud University Medical Centre (NL72784.091.20). Informed consent for secondary use of the data was obtained from all individual participants included in the study.

## Data Availability

The data that support the findings of this study are available from the corresponding author upon reasonable request.

## References

[1] Audenaert E , Audenaert A , De Wilde L , Verdonk R . Defining the shortest path in wrapping algorithms for musculoskeletal modeling. Comput Methods Biomech Biomed Eng. 2005;8(Suppl 1):11.

[2] Beckert M , Crebs D , Nieto M , Gao Y , Albright J . Lateral patellofemoral ligament reconstruction to restore functional capacity in patients previously undergoing lateral retinacular release. World J Clin Cases. 2016;4(8):202–206. 27574606 10.12998/wjcc.v4.i8.202 PMC4983689

[3] Bollier M , Fulkerson J , Cosgarea A , Tanaka M . Technical failure of medial patellofemoral ligament reconstruction. Arthroscopy. 2011;27(8):1153–1159. 21664791 10.1016/j.arthro.2011.02.014

[4] Boot MR , van de Groes SAW , Dunning H , Tanck E , Janssen D . Length changes of the medial patellofemoral ligament during in vivo knee motion: an evaluation using dynamic computed tomography. Am J Sports Med. 2023;51(14):3724–3731. 37960850 10.1177/03635465231205597 PMC10691293

[5] Borbas P , Koch PP , Fucentese SF . Lateral patellofemoral ligament reconstruction using a free gracilis autograft. Orthopedics. 2014;37(7):e665–e668. 24992066 10.3928/01477447-20140626-66

[6] Capkin S , Zeybek G , Ergur I , Kosay C , Kiray A . An anatomic study of the lateral patellofemoral ligament. Acta Orthop Traumatol Turc. 2017;51(1):73–76. 27516002 10.1016/j.aott.2016.07.009 PMC6197417

[7] Chen H , Kluijtmans L , Bakker M , Dunning H , Kang Y , van de Groes S , et al. A robust and semi‐automatic quantitative measurement of patellofemoral instability based on four dimensional computed tomography. Med Eng Phys. 2020;78:29–38. 32115353 10.1016/j.medengphy.2020.01.012

[8] Dejour DH , Mesnard G , Giovannetti de Sanctis E . Updated treatment guidelines for patellar instability: “un menu à la carte”. J Exp Orthop. 2021;8(1):109. 34837157 10.1186/s40634-021-00430-2 PMC8626553

[9] Dunning H , van de Groes SAW , Buckens CF , Prokop M , Verdonschot N , Janssen D . Fully automatic extraction of knee kinematics from dynamic CT imaging; normative tibiofemoral and patellofemoral kinematics of 100 healthy volunteers. Knee. 2023;41:9–17. 36608361 10.1016/j.knee.2022.12.011

[10] Grassi A , Asmonti I , Bignozzi S , Zaffagnini S , Neri MP , Cionfoli C , et al. The sagittal geometry of the trochlear groove could be described as a circle: an intraoperative assessment with navigation. Knee Surg Sports Traumatol Arthrosc. 2021;29(6):1769–1776. 32785759 10.1007/s00167-020-06224-w

[11] Hinckel BB , Yanke AB , Lattermann C . When to add lateral soft tissue balancing. Sports Med Arthrosc. 2019;27(4):e25–e31. 31688535 10.1097/JSA.0000000000000268

[12] Huber C , Zhang Q , Taylor WR , Amis AA , Smith C , Hosseini Nasab SH . Properties and function of the medial patellofemoral ligament: a systematic review. Am J Sports Med. 2020;48(3):754–766. 31091114 10.1177/0363546519841304

[13] Huddleston HP , Chahla J , Gursoy S , Williams BT , Dandu N , Malloy P , et al. A Comprehensive description of the lateral patellofemoral complex: anatomy and anisometry. Am J Sports Med. 2022;50(4):984–993. 35373608 10.1177/03635465221078033

[14] Iranpour F , Merican AM , Baena FRY , Cobb JP , Amis AA . Patellofemoral joint kinematics: the circular path of the patella around the trochlear axis. J Orthop Res. 2010;28(5):589–594. 19950364 10.1002/jor.21051

[15] Iranpour F , Merican AM , Dandachli W , Amis AA , Cobb JP . The geometry of the trochlear groove. Clin Orthop Relat Res. 2010;468(3):782–788. 19915941 10.1007/s11999-009-1156-4 PMC2816780

[16] Jaecker V , Brozat B , Banerjee M , Otchwemah R , Bouillon B , Shafizadeh S . Fluoroscopic control allows for precise tunnel positioning in MPFL reconstruction. Knee Surg Sports Traumatol Arthrosc. 2017;25(9):2688–2694. 25957603 10.1007/s00167-015-3613-9

[17] Kefala V , Ali AA , Hamilton LD , Mannen EM , Shelburne KB . Effects of weight‐bearing on tibiofemoral, patellofemoral, and patellar tendon kinematics in older adults. Front Bioeng Biotechnol. 2022;10:820196. 35497367 10.3389/fbioe.2022.820196 PMC9048742

[18] Kernkamp WA , Wang C , Li C , Hu H , van Arkel ERA , Nelissen RGHH , et al. The medial patellofemoral ligament is a dynamic and anisometric structure: an in vivo study on length changes and isometry. Am J Sports Med. 2019;47(7):1645–1653. 31070936 10.1177/0363546519840278

[19] Klein S , Staring M , Murphy K , Viergever MA , Pluim JP . Elastix: a toolbox for intensity‐based medical image registration. IEEE Trans Med Imaging. 2010;29(1):196–205. 19923044 10.1109/TMI.2009.2035616

[20] Li X , Chen H , Qi X , Dou Q , Member S . H‐DenseUNet: hybrid densely connected unet for liver and tumor segmentation from CT volumes. IEEE Trans Med Imaging. 2018;37(12):2663–2674. 29994201 10.1109/TMI.2018.2845918

[21] Marberry K , Boehm K , Korpi F , Johnson J , Kondrashov P . Anatomical and radiographic characterization of the lateral patellofemoral ligament of the knee. Mol Med. 2020;117(5):469–474. PMC7723147 33311757

[22] Moatshe G , Cram TR , Chahla J , Cinque ME , Godin JA , Laprade RF . Medial patellar instability: treatment and outcomes. Orthop J Sports Med. 2017;5(4):1–6. 10.1177/2325967117699816 PMC5400206 28451613

[23] Nonweiler DE , Delee JC . The diagnosis and treatment of medial subluxation of the patella after lateral retinacular release. Am J Sports Med. 1994;22(5):680–686. 7810793 10.1177/036354659402200517

[24] Parikh SN , Nathan ST , Wall EJ , Eismann EA . Complications of medial patellofemoral ligament reconstruction in young patients. Am J Sports Med. 2013;41(5):1030–1038. 23539043 10.1177/0363546513482085

[25] Powers CM , Ward SR , Fredericson M , Guillet M , Shellock FG . Patellofemoral kinematics during weight‐bearing and non‐weight‐bearing knee extension in persons with lateral subluxation of the patella: a preliminary study. J Orthop Sports Phys Ther. 2003;33(11):677–685. 14669963 10.2519/jospt.2003.33.11.677

[26] Sanchis‐Alfonso V , Montesinos‐Berry E , Ramirez‐Fuentes C , Leal‐Blanquet J , Gelber PE , Monllau JC . Failed medial patellofemoral ligament reconstruction: causes and surgical strategies. World J Orthop. 2017;8(2):115–129. 28251062 10.5312/wjo.v8.i2.115 PMC5314141

[27] Saper MG , Shneider DA . Lateral patellofemoral ligament reconstruction using a quadriceps tendon graft. Arthrosc Tech. 2014;3(4):e445–e448. 25264506 10.1016/j.eats.2014.04.007 PMC4175168

[28] Schöttle PB , Schmeling A , Rosenstiel N , Weiler A . Radiographic landmarks for femoral tunnel placement in medial patellofemoral ligament reconstruction. Am J Sports Med. 2007;35(5):801–804. 17267773 10.1177/0363546506296415

[29] Shah KN , DeFroda SF , Ware JK , Koruprolu SC , Owens BD . Lateral patellofemoral ligament: an anatomic study. Orthop J Sports Med. 2017;5(12):1–6. 10.1177/2325967117741439 PMC5718311 29230426

[30] Song GY , Hong L , Zhang H , Zhang J , Li Y , Feng H . Iatrogenic medial patellar instability following lateral retinacular release of the knee joint. Knee Surg Sports Traumatol Arthrosc. 2016;24(9):2825–2830. 25618277 10.1007/s00167-015-3522-y

[31] Stephen JM , Lumpaopong P , Deehan DJ , Kader D , Amis AA . The medial patellofemoral ligament: location of femoral attachment and length change patterns resulting from anatomic and nonanatomic attachments. Am J Sports Med. 2012;40(8):1871–1879. 22729504 10.1177/0363546512449998

[32] Teitge R , Spak R . Lateral patellofemoral ligament reconstruction. Arthroscopy. 2004;20(9):998–1002. 15525935 10.1016/j.arthro.2004.07.005

